# Predictors of Self-Reported Hand Hygiene Performance among Nurses at Tertiary Care Hospitals in East Coast Malaysia

**DOI:** 10.3390/ijerph18020409

**Published:** 2021-01-07

**Authors:** Mohamad Hazni Abd Rahim, Mohd Ismail Ibrahim, Siti Suraiya Md Noor, Norhana Mohamed Fadzil

**Affiliations:** 1 Department of Community Medicine, School of Medical Sciences, Universiti Sains Malaysia, Kota Bharu 16150, Malaysia; drmohamadhazni@student.usm.my; 2 Department of Microbiology and Parasitology, School of Medical Sciences, Universiti Sains Malaysia, Kota Bharu 16150, Malaysia; ssuraiya@usm.my; 3 Head of Medical Quality Unit, Kelantan State Health Department, Kota Bharu 15590, Malaysia; drhana@moh.gov.my

**Keywords:** predictors, self-reported performance, hand hygiene, nurses, tertiary care

## Abstract

Background: Hand hygiene (HH) is the simplest and most effective way to reduce the incidence of healthcare-associated infections (HCAIs). Methods: This cross-sectional study aimed to determine factors associated with self-reported HH performance among nurses at Kelantan tertiary care hospitals. A sample of 438 registered nurses was selected through a stratified random sampling method. Self-reported HH performance was assessed using a validated WHO self-administered HH knowledge and perception questionnaire for healthcare workers. Results: A multiple linear regression analysis was performed to identify the predictors. The factors that significantly predicted self-reported HH performance among nurses included perception score (beta coefficient (β) = 0.260; 95% CI: 0.200, 0.417; *p* < 0.001), pediatric department (β = −0.104; 95% CI: −9.335, −2.467; *p* < 0.001), and orthopedic department (β = −5.957; 95% CI: −9.539, −0.720; *p* < 0.023), adjusted R^2^ = 0.102; *p* < 0.001. Nurses with a strong perception and belief in HH were more likely to have better HH performance. Compared to pediatric and orthopedic, surgical departments were associated with better self-reported HH performance. Conclusions: This study showed the importance of factors that could improve the intervention’s performance in HH strategy. Lack of perception and HH program intervention in departments engaged in patient care could lead to poor HH practices, thus increasing HCAIs and antimicrobial resistance (AMR).

## 1. Introduction

Globally, thousands of people die every day from infections while receiving healthcare [1]. Healthcare-associated infections (HCAIs) are a major safety concern for both patients and healthcare providers [2] and they continue to escalate at an alarming rate [3,4]. A meta-analysis was published in Lancet in 2011 revealing that the burden of endemic HCAIs in developing countries was 15.5 per 100 patients and 13.9 per 100 patients in Malaysia [5]. In response to HCAIs, the World Health Organization (WHO) launched a patient safety program and multimodal hand hygiene (HH) improvement strategy to establish ways of improving global health and saving lives lost to HCAIs. Participating in HH before and after being with a patient is the single most effective way to prevent the transfer of microorganisms [6].

For all healthcare staff, that are directly or indirectly engaged in patient care, it is important to conduct HH at the right time [7]. Nurses are among the multidisciplinary healthcare professionals that regularly provide bedside care to and are in direct contact with patients. The role of nursing is fundamental to healthcare; nurses in all settings are involved in self-directed and integrated care of people of all ages [8]. Of all healthcare staff, mainly nurses communicate with patients 24 h a day [9] and this contact could pose a greater risk of HCAIs being transmitted to patients. Improving HH performance in healthcare systems will decrease HCAIs by as much as 50 percent [10]. Therefore, nursing strategies play an essential role in preventing HCAIs by ensuring that effective HH practices are followed.

Monitoring HH performance, perceptions, and knowledge is an important part of a three-step process of the HH multimodal improvement strategy in the WHO guidelines [1]. Self-reported HH performance is one of the direct methods of assessing HH compliance. Evaluation of and feedback for HH performance provides healthcare workers with an awareness of improvements available for better HH performance. Studies have shown that the implementation of the WHO HH multimodal improvement strategy can significantly improve HH compliance [7,10,11]. The WHO strongly recommends HH monitoring with feedback to be a consideration as a key performance indicator at the national level [12].

The Malaysian government has acknowledged that patient safety is gaining greater attention than ever before and that many interventions have taken place. There are limited studies conducted in Malaysia to determine the predictors of self-reported HH performance among nurses particularly using the WHO questionnaire. Only one local study focusing on this issue was published seven years ago at one hospital in Klang, West Coast, Malaysia [13]; however, the study was only conducted with ICU nurses and did not include perception variables. HH performance varies depending on certain factors, such as the people involved, the healthcare system, work characteristics, and culture. Therefore, it is difficult to draw a realistic view of HH practices and the factors that impact HH performance of nurses in Malaysia. The current proposed study is useful and important for determining factors associated with self-reported HH performance among nurses. The findings of the study could become a reference point for all relevant stakeholders to apply intervention strategies for improvement.

## 2. Materials and Methods 

### 2.1. Study Setting and Participants

A cross-sectional study was conducted among registered nurses working in tertiary hospitals in Kelantan, East Coast, Malaysia, from 15 December 2019 to 15 February 2020. Tertiary hospitals are specialized advisory centers, usually referred to as primary or secondary medical care, consisting of staff and facilities for special examination and treatment in Malaysia. The four tertiary hospitals included Hospital Raja Perempuan Zainab II, Hospital Universiti Sains Malaysia, Hospital Sultan Ismail Petra, and Hospital Tanah Merah.

The sample size requirement for this study was calculated using two independent mean calculations with the power of study set at 80%, type one error set at 0.05, the mean ratio set as one, and the standard deviation of knowledge scores obtained from a previous study was 0.11 [14]. The minimum sample size required was 424 and, with the additional assumption of a 20% dropout rate, the final number of samples required was 530.

A stratified sampling method was used. Four hospitals were the first strata with the next strata being the types of departments in each hospital. The selection of survey participants from the sample size of the strata in each department was proportionate to the list of nurses available.

Our study included registered nurses working in tertiary hospitals who had at least six months of working experience. The nurses who only did administrative service and assistant nurses were excluded from the study. The self-reported performance was defined as nurses who estimated how frequently they performed HH as recommended by HH guidelines [15]. Variables provided were used to determine the predictors of self-reported HH performance.

### 2.2. Instrumentation

The data was collected using two self-administered questionnaires: the 2009 revision of the WHO hand hygiene knowledge questionnaire for healthcare workers and the WHO perception survey for healthcare workers [16]. In total, 35 questions had to be answered by the participants. The average time to complete the questionnaires was about ten minutes Three domains for the study were adapted from both questionnaires, namely, socio-demographic data (domain A), HH knowledge (domain B), and perception survey (domain C). Domain A consisted of four items of background data and seven work characteristics. Domain B started from Q17 to Q24, for each respondent, the nurse who scored with the correct answers was counted and recorded.

There were 18 items in the questionnaire for domain C, and three items (Q26–Q28) used a 4-point scale. Twelve out of 18 items (Q30–Q34) were rated using a 7-point scale. Score percentages ranged from 0 to 100 percent in Q25, Q29, and Q35. Q25 and Q29 were excluded from the study. Sixteen items were included in the score for perception. For the evaluation of self-reported HH results, Q35 was analyzed separately and score percentages ranged from 0 to 100%.

### 2.3. Statistical Analysis

All 530 questionnaires were distributed to the participants and coded from 1 to 530. The data were entered into Microsoft Excel Office 365 and exported into IBM SPSS version 26 software for analysis. After being entered, the data were analyzed, tested, and cleaned. A preliminary data description was used to detect missing values and check for errors. Missing data for specific and individual items in the study were excluded from the analysis. Simple and multiple linear regression analyses were used to identify predictors of self-reported HH performance. Dummy variables were generated for categorical variables for analysis.

Simple linear regression was used to select the preliminary variables with a *p*-value less than 0.05, or any clinically relevant or important variable was selected for multiple linear regression analysis. These variables were analyzed using forward, backward, and stepwise methods. All possible two-way interactions and multicollinearity were checked.

Then, the final model was obtained after checking model fitness and assumptions. The value of adjusted R^2^ in the modeling showed that the variation of predictors is explained by self-reported HH performance. The level of significance was set at a *p*-value less than 0.05.

### 2.4. Ethical Consideration

Ethical clearance was obtained from the Universiti Sains Malaysia Human Etiquette Committee, (JEPeM-USM) with JEPeM code: USM/JEPeM/19100595 on 5 December 2019 and from the National Medical Research Registry (NMRR) on 16 January 2020 with reference number: NMRR-19-3365-51286. The data were purely restricted, and only the author and supervisor had access to the information. Subsequently, analyses and publications were made without the names of the respondents listed.

## 3. Results

### 3.1. Socio-Demographic Characteristics of Participants

A total of 530 questionnaire surveys were distributed with a response rate of 87.5% from 438 questionnaires fully completed. The mean (SD) age of nurses was 38.4 (7.314) years and the majority were female (93.2%). Most of the participants had diplomas without post basic. A summary of the socio-demographic characteristics of the participants is shown in Table 1.

### 3.2. Work Characteristics of Participants

The average clinical work experience of the participants was 14.93 (6.913) years. Almost all participants worked as staff nurses (90.6%) and non-infection control nurses (95.2%). Remarkably, most participants reported receiving formal HH training in the last three years at 91.8% and routinely used alcohol-based cleaner for HH at 97.5%. Table 2 below illustrates the work characteristics of the participants.

### 3.3. Predictors of Self-Reported HH Performance

#### 3.3.1. Simple Linear Regression

To understand factors that could predict the success of self-reported HH performance among nurses in tertiary care hospitals, all the variables of the study (socio-demographics, work characteristics, knowledge, and perception scores) were entered into a regression analysis. The results showed there were five significant linear relationships between self-reported HH performance with the orthopedic department (*p* = 0.039), pediatric department (*p* = 0.025), obstetrics and gynecology (O&G) department (*p* = 0.006), state general hospital (*p* = 0.033), and perception score (*p* < 0.001). A summary of the findings is illustrated in Table 3.

#### 3.3.2. Multiple Linear Regression

A stepwise multiple regression analysis was employed to identify the predictors. Five factors were included in the prediction model, but only three factors were significant at predicting the self-reported HH performance among nurses. The final regression model was calculated as follows: 67.876 + 0.260 (perception score), −0.104 (pediatric department), and −0.155 (orthopedic department). The model accounted for 10.2% of the variance of the self-reported HH performance, R^2^ = 0.110, adjusted R^2^ = 0.102. As presented in Table 4, the pediatric department, orthopedic department, and perception scores were identified as predictors of self-reported HH performance among the participants.

## 4. Discussion

HCAIs lead to prolonged hospital stays, long-term disabilities, microorganisms’ resistance to treatment, the additional financial burden from increased management costs, and unnecessary deaths. Among healthcare workers, nurses are mostly in contact with patients 24 h a day [9] as main providers of physical care. Due to their regular interaction with patients, the proper implementation of HH by nurses plays a particularly important role in the prevention of HCAIs and the latest HH recommendations should be followed [17]. When factors associated with HH can be identified, meaningful and sustainable interventions can be designed to drive HH compliance toward 100% with an associated decrease in HCAIs [18,19,20].

In this study, most of the respondents were female (93.2%), matching previous studies in Sibu, Malaysia [21], and indicating that the nursing profession in Malaysia is predominantly comprised of females. The average age was 38.43 ± 7.314 years, which was relatively higher than previous studies among nurses, such as 31.2 ± 7.3 years [17], 32.7 ± 4.6 years [22], and 29.4 ± 5 years [23], reflecting most of the young nurses in the hospital. More clinical work experience had a greater influence on HH practice [13], which was closely related to the age of respondents. On average, the work experience of participants was 14.93 ± 6.913 years higher than previous studies, such as 6.94 ± 5.61 years [9], 14.2 ± 10.2 years [24], and 10.12 ± 13.50 years [25]. Almost all nurses, 402 or 97.5%, received HH training within the last three years, compared to lower percentages of other studies, such as 85.2% [21] and 75% [26], which possibly implied that the infection control team and top management of the hospitals implemented good training program coverage among nurses.

Consistent with other studies [17,26,27], the current study illustrated that the perception score had a significant linear relationship to self-reported HH performance, possibly because nurses with a strong perception and belief in HH were more likely to exhibit good HH behavior. A clear understanding of the impact of HCAIs on patients, a high level of personal effort in HH, pressure from subjective norm, and the priority that managers and organizations have put on HH are possible influences on the nurses’ perception scores. This is consistent with the study by White et al. [27] that found nurses were influenced by pressure from others, the nurses’ perceptions of organizational priorities [26], the personal effort required in HH, and the impact of HCAIs. Awareness of the importance that HH plays in the prevention of HCAIs was also an essential association with HH practices [17].

A cross multi-center study conducted by Hyang Soon O in 2018, among registered nurses at community-based hospitals in the Republic of Korea found that perception, attitude, and role model were found to be significant personal predictors of HH performance. Perceptions have been shown to be significant predictors of nurses’ HH intentions and adherence [28].

The staff pediatric department, with 0.104, had lower self-reported HH performance compared to the surgical department, which suggested that the surgical department was associated with better self-reported HH performance. This is consistent with a study conducted by Lee et al. [29] which showed that the surgical and medical departments adhered to HH more than other specialty departments. This may be explained by the surgical department’s emphasis on the importance of HH as a compulsory and standard operating procedure in the operation theatre [30]. Regularly performing HH as a daily routine will influence the habits of nurses to practice HH. Possible explanations for lower levels of HH in pediatric departments are busy schedules and dealing with acute childhood illnesses. Dealing with sick children has many challenges because they are more fragile and deteriorate faster than adult patients. Similar to [31], patient burden responsibilities, hectic schedules, and pediatric emergencies influenced the need to balance priorities with HH practices.

Other significant findings demonstrated by orthopedic departments, such as less concentration on HH campaigns and less tracking enforcement, may indicate why orthopedics has lower self-reported HH performance compared to surgical departments. A previous study by Gupta et al. [32] indicated that HCAI cases were lower in the female surgery department from 2012 to 2016 than in the orthopedic department. To better prioritize HH in all departments, one suggestion was to engage in a competition and award a prize for the best performing department in an HH campaign. This may motivate top management as a key performance indicator for the department. In a friendly competition setting, a multimodal intervention program significantly increases compliance with HH [33].

The results of this study showed no significant association with self-reported HH performance when receiving HH training within three years. This finding does not support previous studies that showed training received had a significant association with HH performance [34,35,36,37]. Training nurses once every three years may not be enough, since motivation among nurses is generally associated with maintaining a level of awareness about the issue. Previous study suggested that having training repeated helps with HH compliance since adherence tends to decline two years after the initial training campaign [34]. Training not only improved knowledge, but repeated training was part of a campaign for awareness and motivation in a multimodal HH strategy.

## 5. Limitations

The self-administered questionnaire may result in information bias by giving the participants the opportunity to discuss answers with colleagues. It also imposes peer pressure on top management who may be fearful that truthful answers could reflect badly on the poor performance of their organization. Evaluations of the self-reported performance, instead of the actual HH compliance via a gold standard WHO direct observation method [1], may also be considered a limitation.

Self-reported HH compliance measurement performance is an acceptable, timely, and low-cost observational study alternative that can be measured by Human Resources. However, it should not be interpreted to imitate the reality of HH compliance as it might create overestimation in the measurement [26]. Another study conducted by [38] on healthcare workers’ perceptions of self-reported HH performance stated that a much higher score might be considered an overestimation of the observed method. Despite these limitations, this research provided valuable input into developing strategies for HH intervention in hospitals in East Coast, Malaysia.

## 6. Conclusions

The current study demonstrated the importance of determining predictors that could optimize the effectiveness of HH intervention strategies among nurses at tertiary care hospitals in East Coast, Malaysia. Healthcare personnel responsible for organizational policies should concentrate on improving the perception and belief in HH, which has shown significant results for self-reported performance of HH among nurses. Pediatric and orthopedic departments were found to be significant predictors of low self-reported HH performance; this should be taken into account by healthcare professionals when implementing programs for HH intervention. It is recommended that future research comparing all healthcare workers at every level of care should be considered at the national level.

## Figures and Tables

**Table 1:** Socio-demographics of the participants (*n* = 438).

Variables	*n* (%)	Mean (±SD)
Gender		
Female	408 (93.2)	
Male	30 (6.8)	
Age		38.43 ± 7.31
Marital status		
Married	393 (89.7)	
Unmarried	45 (10.3)	
Education level		
O level	24 (5.5)	
Diploma	353 (80.6)	
Master/PhD	5 (1.1)	
Diploma with post basic	56 (12.8)	

**Table 2:** Work characteristics of participants (*n* = 438).

Variables	*n* (%)	Mean (±SD)
Work experience		14.93 ± 6.91
Infection control nurse (ICN)		
ICN	21 (4.8)	
Non-ICN	417 (95.2)	
Hospital		
State general hospital	194 (44.3)	
Teaching hospital	165 (37.7)	
District specialist hospital	79 (18.0)	
Positions		
Nurse	397 (90.6)	
Sister (charge nurse)	36 (8.2)	
Matron (head nurse)	5 (1.1)	
Department		
Internal medicine	53 (12.1)	
Surgery	42 (9.6)	
Intensive care unit	83 (18.9)	
Orthopedics	28 (6.4)	
Emergency	7 (1.6)	
Pediatrics	49 (11.2)	
Obstetrics and Gynecology (O&G)	67 (15.3)	
Operation room	24 (5.5)	
Others (outpatient, rehabilitation, etc.)	85 (19.4)	
Received Hand Hygiene (HH) training within the last three years		
Yes	402 (91.8)	
No	36 (8.2)	
Routinely use hand rub for HH		
Yes	427 (97.5)	
No	11 (2.5)	

**Table 3:** Factors associated with self-reported HH performance using simple linear regression analysis (*n* = 438).

Variables	β (Unstandardized)	SE	95% CI	*p*-Value
Age	0.077	0.079	−0.077, −0.232	0.326
Gender				
Male	0			
Female	1.083	2.277	−3.392, 5.558	0.635
Marital Status				
No	0			
Yes	0.062	1.895	−3.662, 3.786	0.974
Position				
Matron	0			
Sister	−7.833	5.740	−19.115, 3.448	0.173
Nurse	−7.444	5.412	−18.082, 3.193	0.170
Education level				
O level	0			
Diploma	−0.488	2.491	−5.384, 4.408	0.845
Master/PhD	2.288	5.896	−9.300, 13.876	0.698
Diploma with post basic	2.056	2.895	−3.634, 7.745	0.478
Work experience	0.123	0.083	−0.041, 0.286	0.141
Type of department				
Surgery	0			
Internal medicine	−3.388	2.434	−8.171, 1.396	0.165
Intensive Care Unit (ICU)	−0.061	2.231	−4.445, 4.324	0.978
Orthopedic	−5.957	2.874	−11.606, −0.308	0.039
Emergency	1.936	4.809	−7.517, 11.389	0.688
Pediatric	−5.574	2.477	−10.443, −0.706	0.025
Obstetrics and Gynecology	−8.308	3.014	−14.233, −2.383	0.006
Operation room	1.250	2.222	−3.117, 5.617	0.574
Type of hospital				
District hospital with specialist	0			
State hospital	3.420	1.600	0.275, 6.565	0.033
Teaching hospital	2.737	1.640	−0.487, 5.961	0.096
Infection control nurse (ICN)				
Non−ICN	0			
ICN	4.162	2.685	−1.116, 9.440	0.122
Received training in last three years				
No	0			
Yes	−0.699	2.094	−4.815, 3.418	0.739
Routinely use hand rub for HH				
No	0			
Yes	4.261	3.671	−2.954, 11.476	0.246
Knowledge score	0.372	0.293	−0.206, 0.947	0.207
Perception score	0.320	0.055	0.212, 0.427	<0.001

**Table 4:** Predictors of self-reported HH performance using multiple linear regression analysis (*n* = 438).

Variables	β (Unstandardized) (95% CI)	*p*-Value	β (Standardized) (95% CI)	*p*-Value
Intercept	67.876	<0.001		<0.001
Perception score	0.320		0.260	<0.001
	(0.212, 0.427)		(0.200, 0.417)	
Type of department				
Surgery	0		0	
Pediatric	−5.574 (−10.443, −0.706)	0.025	−0.104 (−9.335, −2.467)	0.001
Orthopedic	−5.957 (−11.606, −0.308)	0.039	−0.155 (−9.539, −0.720)	0.023

Stepwise multiple linear regression method applied. The model fits reasonably well. The model has met multiple linear regression assumptions. No independent variable interactions and no multicollinearity. Coefficient of determinants, R^2^ (adjusted) = 10.2%.

## Data Availability

Data sharing is not applicable to this article.
